# Potential Role of Traditional Birth Attendants in Neonatal Healthcare in Rural Southern Nepal

**DOI:** 10.3329/jhpn.v27i1.3317

**Published:** 2009-02

**Authors:** Tina Y. Falle, Luke C. Mullany, Nandita Thatte, Subarna K. Khatry, Steven C. LeClerq, Gary L. Darmstadt, Joanne Katz, James M. Tielsch

**Affiliations:** ^1^ Department of International Health, Johns Hopkins Bloomberg School of Public Health, 615 North Wolfe Street, E8646, Baltimore, MD 21205, USA; ^2^ Nepal Nutrition Intervention Project–Sarlahi, Kathmandu, Nepal

**Keywords:** Community-based interventions, Knowledge, attitudes, practices, Neonatal mortality, Traditional birth attendants, Nepal

## Abstract

The potential for traditional birth attendants (TBAs) to improve neonatal health outcomes has largely been overlooked during the current debate regarding the role of TBAs in improving maternal health. Randomly-selected TBAs (n=93) were interviewed to gain a more thorough understanding of their knowledge, attitudes, and practices regarding maternal and newborn care. Practices, such as using a clean cord-cutting instrument (89%) and hand-washing before delivery (74%), were common. Other beneficial practices, such as thermal care, were low. Trained TBAs were more likely to wash hands with soap before delivery, use a clean delivery-kit, and advise feeding colostrum. Although mustard oil massage was a universal practice, 52% of the TBAs indicated their willingness to consider alternative oils. Low-cost, evidence-based interventions for improving neonatal outcomes might be implemented by TBAs in this setting where most births take place in the home and neonatal mortality risk is high. Continuing efforts to define the role of TBAs may benefit from an emphasis on their potential as active promoters of essential newborn care.

## INTRODUCTION

Formative research to understand local beliefs, practices and their determinants has been identified as a major gap to developing effective perinatal and neonatal interventions in communities of developing countries (1,2). Traditional birth attendants (TBAs) play an important role in settings where most births take place in the home (3) and, in Asia, constitute the largest single group of birth attendants (41% of births) (4). The World Health Organization (WHO) defines TBA as “a person who assists the mother during childbirth and initially acquired her skills by delivering babies herself or through apprenticeship to other TBAs” (5). TBAs are integral members of their communities and provide an important window to local customs, traditions, and perceptions regarding childbirth and newborn care (6,7).

The role of TBAs in improving maternal health has been heavily debated, especially in the context of a renewed focus on Millennium Development Goal (MDG) 5. While trained TBAs are not considered skilled birth attendants (SBAs), their potential contribution has been recognized in diagnosing labour, ensuring clean delivery, detecting and referring maternal complications, providing hygienic cord-care and ensuring warmth of the newborn, supporting early exclusive breastfeeding, and providing advice on a number of topics (8,9). Other interventions, such as routine administration of misoprostol for the prevention of postpartum haemorrhage, while having been successfully administered by community-based auxiliary nurse midwives (10) and by TBAs (11), need further research and evaluation.

In Nepal, 81% of births take place in the home (12). Despite recent efforts to scale up the coverage of skilled attendance at birth, leading to an increase from 9% to 19% over the last decade (12,13), the country is still far from attaining universal coverage. Neonatal mortality is high (33/1,000 livebirths) and accounts for more than two-thirds of infant deaths (12). The proportion of births assisted by TBAs (*Sudeni*, 19%) has remained steady since 1995 (12,13), and TBA attendance in the *terai*, or southern plains region of Nepal, is even higher (31%) (12). Women giving birth without the assistance of a skilled attendant or a TBA are normally assisted by family members, neighbours, or deliver alone. The low access to skilled care, high risk of neonatal death, and integral role of TBAs and other non-skilled care providers in delivery and neonatal care highlight the need to explore alternative, low-cost, efficacious community-based interventions outside health facilities, especially for newborns.

Due to the varied nature, function, knowledge, and experience of TBAs in different settings, it is important to gain a more thorough understanding of the range of local practices and their prevalence that may lead to identification of behaviour-change interventions to improve neonatal health and survival. We assessed the knowledge, attitudes, and practices (KAPs) of TBAs in Sarlahi district of southern Nepal using a representative sample of trained and untrained TBAs from the two major ethnic groups in the region.

## MATERIALS AND METHODS

The study was conducted at the field site of the Nepal Nutrition Intervention Project (NNIPS) in Sarlahi district in the southern plains (*terai*) of Nepal. The population of 230,000 in this area lives mainly in rural farming communities and consists of two broadly-defined ethnic groups: Maithili-speaking ‘Madeshi’ (originating from the southern plains region and comprising mostly lower castes) and mainly Nepali-speaking ‘Pahadi’ (originating from the hills and comprising mostly higher castes, i.e. Brahmin and Chettri). While there are some differences in newborn-care practices between these two main population groups in this community, most behaviours are similar. For example, Madeshis are less likely to give colostrum (77.5% vs 90.8%) or to initiate breastfeeding within 24 hours (41.9% vs 92.9%) but delayed bathing, oil massage, and hand-washing practices are similar (14-16).

The survey instrument was developed from formative research conducted in this site in 2003-2004. To assess and distinguish actual practices from knowledge, the instrument included questions about the practices of TBAs (asking what TBAs did during their most recent delivery) and a separate section on general opinions and/or knowledge of TBAs (asking what ‘should’ be done). The instrument contained sections on the TBAs' background, training, antenatal and delivery practices, knowledge of complications, newborn-care practices, post-delivery care, payment, and attitudes and perceptions about their profession and newborn care.

Attendants (*chamains*) who only perform a subset of TBA tasks, such as cutting the cord, were excluded from our study population. As prior information on TBAs was not readily available, the workers of the NNIPS project constructed a list of all trained and untrained TBAs in the study area as reported by community leaders. Three of 495 TBAs (344 Madeshi and 151 Pahadi) were excluded from selection for inaccessibility to their working area. A simple random sample of 75 TBAs from each of the two main ethnic groups (Pahadi and Madeshi) was selected from among the remaining 492 TBAs. The sample size was largely based on time and staffing constraints, and the Pahadi TBAs were oversampled for convenience and to achieve balance in group comparisons. TBAs were eligible for inclusion if they reported delivering at least one baby within the three months prior to recruitment.

The workers fluent in the local language of the project area conducted interviews in the home of the TBA, and the interview lasted approximately one hour. Verbal informed consent was obtained prior to the interview, and TBAs received three locally-manufactured clean delivery-kits for their participation.

Data were analyzed using the Stata software (version 9.2) (Stata Corp LP, College Station, Texas). Comparisons between Pahadi and Madeshi, and trained and untrained TBAs were done using Fisher's exact test. The cut-off level for statistical significance was p<0.05. For some questions, the summation of response proportions exceeded 100%, as multiple answer choices were possible. The Nepal Health Research Council and the Johns Hopkins Bloomberg School of Public Health Committee on Human Research reviewed and approved the study procedures.

## RESULTS

### Background of TBAs

Of 150 TBAs initially identified, three were not met after at least three attempts, and 19 Madeshi and 35 Pahadi women had no recent deliveries (within the previous 3 months). Interviews were completed for the remaining 93 female TBAs (54 Madeshi and 39 Pahadi aged 25-73 years (mean age 47.1 years). The vast majority (71%) worked in agriculture and were illiterate (74%). In the preceding year, about one-third (29%) of the TBAs reported delivering 1-5 baby(ies), 33% between 6 and 10 babies, and 38% more than 10 babies. The Pahadi TBAs were more likely to be literate and from higher castes (Brahmin/Chettri). Table 1 provides further information on background characteristics of the TBAs.

**Table 1.** Background characteristics of TBAs by ethnicity

Variable	Madeshi (plains) (n=54, 58%)		Pahadi (hills) (n=39, 42%)		All (n=93)		p value
	No.	%	No.	%	No.	%	
Caste							<0.0001
Brahmin	0	0	13	33.3	13	13.9	
Chettri	2	3.7	7	17.9	9	9.6	
Vaiysha	16	29.6	17	43.6	33	35.4	
Sudra	30	55.6	2	5.1	32	34.4	
Muslim	6	11.1	0	0	6	6.4	
Age (years)							
25-34	8	14.8	6	15.4	14	15.0	
35-44	17	31.5	11	28.2	28	30.1	
45-54	13	24.1	8	20.5	21	22.6	
55-64	11	20.4	10	25.6	21	22.6	
65-74	5	9.3	4	10.2	9	9.7	
Median age (years)	45		48		47		
Literate	4	7.4	20	51.3	24	25.8	<0.0001
Trained	17	31.5	19	48.7	36	38.7	
Trained and literate	2	3.7	12	30.8	14	15.0	<0.01
Experience (years)							
<5	10	18.5	5	12.8	15	16.1	
6-15	19	35.2	18	46.1	37	39.8	
>15	25	46.3	16	41.0	41	44.1	
Median experience (years)	15		14		15		
Regular work beyond TBA practice							
Agriculture, field work	37	68.5	29	74.4	66	71.0	
Caretaker, home-maker	32	59.3	23	59.0	55	59.1	
Day labourer/wages	10	18.5	2	5.1	12	12.9	<0.1
Voluntary work	2	3.7	9	23.1	11	11.8	<0.01
Government/non-government service	2	3.7	5	12.8	7	7.5	
Trade/business	2	3.7	2	5.1	4	4.3	
Number of babies delivered in the last 12 months							
1-5	14	26.4	13	33.3	27	29.3	
6-10	15	28.3	15	38.5	30	32.6	
11-20	14	26.4	7	17.9	21	22.8	
21-50	9	17.0	4	20.3	13	14.1	
51 or more	1	1.9	0		1	1.1	

TBA=Traditional birth attendant

Thirty-six (39%) trained TBAs had their training from health posts or health centres (42%), NGO/INGOs (39%), the district public-health office (8%), or a hospital (8%). The mean duration since the last training was 12 months, and the average training duration was three days; specific details on the content of these training were not collected.

### Practices of TBAs

The first contact of 47% of the TBAs with the pregnant woman occurred more than one month before delivery, about a quarter (28%) within a month of delivery, and another quarter on the day of birth. The TBAs commonly reported delivering the baby (90%), placenta (84%), and cutting the cord (64%) in their most recent delivery. The Madeshi TBAs were less likely than the Padadi TBAs to cut the cord; among Madeshi communities, *chamains*—women of a lower subcaste—were sometimes called to the household to specifically cut the cord and wash the newborn.

During antenatal visits, the TBAs provided advice on diet and nutrition (61%), immunizations (34%), and reducing the workload during pregnancy (21%). More than two-thirds (69%) of the TBAs reported advising on tetanus toxoid (TT) immunizations. Of those, 57% suggested three vaccinations during pregnancy, and 37% suggested two vaccinations. Given the scenario that a pregnancy was perceived to be a threat to the woman's health, almost three-fourths (73%) of the TBAs stated that they would refer her to a clinic, 9% would advise her to eat nutritious food, 8% would advise getting an abortion, and 8% would not do anything.

Eighty-five percent of the TBAs provided postnatal care and advice. Of these, 57% returned within 24 hours after delivery to provide postnatal care to the mother and the baby. Oil massage of the baby (68%) or the mother (52%), and bathing the baby (46%) were the most frequently-reported specific examples of care provided. Thirty-seven of the TBAs provided postpartum advice to the mother regarding nutrition. Information on immunizations, medical care, and/or family planning was more often provided by the Pahadi TBAs (39%) than by the Madeshi TBAs (13%, p=0.009).

### Clean delivery practices

Washing hands with soap prior to delivery was common (74%) among both the ethnic groups, yet varied substantially by training status: 89% of trained TBAs vs 65% of untrained TBAs reported washing hands before delivery (p=0.01). The use of a new blade to cut the cord was reported in 89% of livebirths across training and ethnic groups while the remaining TBAs (11%) used scissors. Cutting the cord after delivery of the placenta was an almost universal practice (99%). The vast majority (88%) of the TBAs reported that the cord was cut more than five minutes after birth; 22% and 12% cut the cord more than 30 and 60 minutes after birth respectively.

About 27% of the TBAs reported the immediate application of a substance to the umbilical cord. Of all substances, mustard oil (64%) was the most common topical application, followed by substances, such as saliva (16%) or antiseptics (12%); information on specific antiseptic was not collected. The Pahadi TBAs were more likely to use a topical cord application than the Madeshi TBAs (40% vs 17% respectively, p=0.02).

Almost half (47%) of the TBAs used a clean delivery-kit in their most recent delivery. The use of the kit was significantly higher (p<0.02) for trained (64%), literate (75%) and Pahadi (64%) TBAs. About half (52%) of kit-users described the correct use of all items in the kit in their most recent delivery, and this was similar across training status and ethnic group.

### Thermal care

Immediately after delivery, 75% of the TBAs placed the baby on the floor. Only 16% reported that the baby was wiped and/or dried, and 28% wrapped the baby within the first three minutes after birth. Evidently, this delay was due to the practice of waiting until after delivery of the placenta to care directly for the baby, although some TBAs stated that the baby was wiped (32%) and/or wrapped (43%) before the placenta was delivered. The TBAs' knowledge of immediate drying (28%) and wrapping (37%) was slightly higher than actual practice. Early bathing (within 1 hour after birth) was reported for the most recent delivery by more than half (53%) of the TBAs, and a similar proportion (56%) of the TBAs also recommended this practice. Only 19% of babies during previously-reported deliveries were washed 24 hours or later after birth. Although no difference was detected by training status, literate TBAs were more likely to delay bathing until after 24 hours (54%) compared to illiterate TBAs (7%, p<0.0001).

### Breastfeeding

About 62% of the TBAs recommended initiating breastfeeding within one hour of birth. This was higher for literate TBAs (83% vs 55%, p=0.03) but similar by ethnicity and training status. However, in actual reported recent practice, the early initiation of breastfeeding (within 1 hour) was only 28% for the Madeshi and 54% for the Pahadi TBAs (p=0.02). Later initiation (24 or more hours postpartum) was reported by 22% of the Madeshi vs 3% of the Pahadi TBAs (p=0.01) (Table 2). Most (97%) Pahadi TBAs recommended feeding colostrum compared to three-fourths (75%) of the Madeshi TBAs (p=0.003). All trained and literate TBAs gave advice on feeding colostrum compared to 78% of untrained (p=0.001) and 75% of illiterate TBAs (p=0.02). This advice was most commonly provided because the TBAs reported that colostrum benefits the overall health of the baby (70%) or boosts the immune system of the baby (37%). Those advising against colostrum (15%) suggested that colostrum was ‘thick and unhealthy’ (57%) or that it would cause diarrhoea (21%).

**Table 2.** Time to initiation of breastfeeding after birth

Time to initiation	Practice (reported initiation of breastfeeding in most recent delivery)			Knowledge (TBAs' recommended initiation of breastfeeding)		
	Trained (n=33) (%)	Untrained (n=54) (%)	All (n=87) (%)	Trained (n=36) (%)	Untrained (n=56) (%)	All (n=92) (%)
Immediately (<10 minutes)	6.1	5.6	5.8	13.9	10.7	12.0
11 minutes to 1 hour	45.4	25.9	33.3	58.3	46.4	51.1
2-11 hours	39.4	44.4	42.5	19.4	30.4	26.1
12-23 hours	6.1	3.7	4.6	2.8	0	1.1
24-47 hours	3.0	7.4	5.8	5.6	10.7	8.7
48-71 hours	0	9.3	5.8	0	1.8	1.1
72 or more hours	0	3.7	2.3	0	0	0

TBAs=Traditional birth attendants

### Oil massage

Baby massage with mustard oil was a universal practice (99%) among recent deliveries. Mustard oil was considered to be superior to other oils because of its ‘hot’ properties (i.e. to keep the baby warm, 60%) and its perceived benefit to the health of the baby (55%). The priority given to mustard oil was not due to objectionable properties of other oils, e.g. stickiness, odour. In fact, more than half (52%) of the TBAs were open to using an alternative oil; those suggested by the TBAs included baby oil (26%), yellow mustard oil (13%), and sunflower oil (13%). Also, 17% of the TBAs who were accepting other oils suggested using any oil currently in the household. In general, the availability of different types of oils was not considered a limitation in their use.

### Complications for newborns

For babies not breathing after birth, traditional care practices, such as milking the cord to ‘send the breath to the baby’, are still widely practised. The use of equipment, such as bag/tube and mask for resuscitation, was not reported. In this sample, most (90%) TBAs recognized poor nutrition as the primary risk factor associated with low-birthweight babies. Other factors mentioned were illness of the mother (20%), continued heavy work during pregnancy (12%), small size of the mother (10%), and prematurity (10%). Signs that a baby requires extra attention included ‘weak’ (67%), light weight, or appearing abnormally small (57%), and difficulty in suckling (27%). A ‘weak’ baby or one feeling/looking small was recognized as indicative of a baby at risk of dying; other reported signs included respiratory distress (37%) and prematurity (30%).

### Referral for maternal complications

When a complication during delivery could not be managed, the TBAs reported that they would send the woman to a clinic/hospital (75%) and/or would call a doctor to the house (41%). In actual practice, however, only 53% of the TBAs reported ever having referred a woman with delivery-related complications to a health facility or a doctor. Whether or not the TBAs had ever referred did not depend on their training status, ethnic background, years of experience, or literacy. About 61% of trained TBAs cited excessive bleeding as a reason for referral compared to 39% of untrained TBAs (p=0.05). Excessive bleeding was defined subjectively by the TBAs—they generally ‘knew by seeing’ (83%). Also, 14% of the TBAs counted the number of pieces of blood-soaked cloth as an indicator of excessive bleeding, although the number of pieces ranged from 2 to 6.

## DISCUSSION

Data presented here provide insight into common care practices for mothers and newborns in the community, in addition to practices, perceptions, and knowledge of the TBAs themselves. Understanding these practices facilitates exploration of the role that TBAs or other minimally-trained caretakers in the community might play in the transition to skilled care.

Some clean delivery practices, such as the use of a clean cord-cutting instrument, were very high overall, and others, such as hand-washing, were significantly higher among trained TBAs. The use of a home delivery-kit, practised during the most recent delivery by 47% of the TBAs in our sample, was more than twice as high as the national rate of 18% (12) but is likely partially due to the promotion of the kits during prior studies (14,17). The kits produced in Nepal (18) have been used for promoting birth-preparedness (12) and clean delivery. However, persisting misconceptions regarding the correct use of items, such as soap (perceived use for bathing the baby, potentially affecting early bathing practices), highlight the importance of the need to re-evaluate strategies for effectively communicating the appropriate use of the kit. If promotion of such kits continues, or incorporation of further low-cost interventions into these kits are considered, programmes will require substantial efforts to improve behaviour-change communications about the proper use of items in the kit to recipients, including TBAs, mothers, and other potential users. Such strategies might include re-evaluation and redesign of pictorial instructions and ensuring standardization and appropriateness of messages provided at the point of sale or distribution.

We found that knowledge and practices of simple methods for keeping a baby warm and preventing hypothermia, such as immediate drying and wrapping, were lacking. The high prevalence of early bathing (<6 hours), a potentially-harmful practice, is comparable with findings reported previously from similar settings (19-21) and may be amenable to community-level mobilization and education (21,22).

Breastfeeding is a standard practice in Nepal, although early and exclusive breastfeeding continues to remain a challenge (19,22). Efforts to promote colostrum and early and exclusive breastfeeding, especially in the Madeshi community, could be of great benefit given the significantly lower levels of knowledge and practice and higher rates of neonatal mortality in these communities. Initiation of breastfeeding within the first hour or first day of life has been associated with a substantially-lower risk of neonatal mortality (16,23), and TBAs could potentially play an important supportive role in the promotion of improved breastfeeding practices.

The common practice of newborn massage with mustard oil has been reported elsewhere in this region (24-26). Mustard oil is of questionable safety for newborns (27) and could be replaced by oils that have been shown to enhance skin-barrier function and reduce mortality, such as sunflower oil (28-30). The TBAs in this setting indicated their willingness to consider alternatives to mustard oil, confirming previous research on attitudes towards alternative oils in the general population (26) and suggesting the potential for development and testing of community-based interventions.

Although the TBAs indicated their willingness to refer, clear decision-making guidelines to know when they cannot ‘manage’ a complication appear to be lacking. The low proportion of TBAs who had ever referred to a health facility may also be due to a lack of true referral options in the study area. As TBAs can play an important role in strengthening links between the community members and the formal health system, knowledge of danger-signs is urgently needed to prevent delays in referring complications. We found that the TBAs lacked a formal methodology to assess postpartum blood loss as 83% stated that they knew excessive bleeding when they ‘saw’ it. Researchers in Tanzania found that two *kanga* (standard-size cotton cloth used by women in East Africa) soaked in blood represented loss of slightly more than 500 mL of blood and used this threshold in training of TBAs (31,32). Opportunities to establish a similar measurement tool using cotton *saris* in South Asia could be further explored, especially since some TBAs already reported counting pieces of blood-soaked cloth.

This study relied solely on self-reported information of TBAs, although there was little incentive to report misinformation, and we attempted to explore the differences between knowledge and practices by specifically asking about practices during the most recent delivery. This effort is limited by the degree to which the most recent delivery represents ‘normal’ practice for an individual TBA. The binary variable indicating training status is non-specific to the range and quality of actual training received, which likely varied substantially within the ‘trained’ group. The instrument was lengthy, and some interviews extended beyond two hours, which may have led to a loss of concentration or fatigue on the part of the respondents.

Given the continued importance of TBAs in many communities, engagement with them and redefinition of their roles may allow for meaningful contributions to maternal and particularly to neonatal health (33,34). A review of 15 maternal mortality interventions using TBAs and village midwives found that the most successful ones shared the characteristic of embedding TBAs in a multi-sectoral intervention with ensured access to emergency obstetric care (35). A meta-analysis of the impact of TBA training found a small but significant decrease in perinatal mortality (8%) and neonatal mortality due to birth asphyxia (11%) (36). A randomized trial of the impact of a three-day TBA training programme in Pakistan demonstrated significant reductions in perinatal (odds ratio [OR]=0.70, 95% CI 0.59-0.82) and neonatal mortality (OR=0.71, 95% CI 0.62-0.83) and improved rates of referral compared to control areas (37), illustrating the potential of integrating TBAs into the local health system.

In this community and other similar low-resource settings, how can the existing networks of TBAs be strengthened to improve neonatal outcomes? We found that the TBAs were eager to receive training (56%, unprompted), and two-thirds (67%) stated that they would need training to improve neonatal health in their communities. Given that the national neonatal health strategy of Nepal supports evidence-based neonatal care at all levels, including home and community levels (38), TBAs could play an effective role as active promoters of essential neonatal care in their communities by promoting effective care, such as clean delivery and cord-care, immediate and exclusive breastfeeding, immediate drying and wrapping, and delayed bathing to other birth attendants from the family (Table 3). The most active TBAs might be recruited to receive training on both improved practices and promote improved practices among the largest group of care providers in this community: family members. TBAs could ease referrals when complications arise, increasing facility-based care-seeking and access to services.

**Table 3.** List of potential interventions for improving neonatal health involving TBAs

Encouraging healthy behaviours by promotion of/training in	
Prevention of tetanus	Tetanus coverage for every pregnant woman in TBAs' areas
	Encouraging TBAs to get to know all pregnant women in her area and inform them about TT vaccination
Preventing delays	Planning transport to facility and other emergency arrangements ahead of time, in case complications arise
Clean delivery	Washing hands of attendant(s) with soap before and after delivery
	Using a new or clean blade and surface to cut the umbilical cord
	Inform on the importance of the ‘5 cleans’ to emphasize the importance of cleanliness during delivery in the home
	Proper use of clean delivery-kit, educate on intended use of every item in the kit
Thermal care	Immediate drying and wrapping of the baby, even if the cord has not been cut
	Handing the newborn to the mother or another family member immediately after birth
	Encouraging skin-to-skin care with the mother
	Delayed bathing until at least 6 hours after birth, preferably waiting until second day: inform on the possibility of negative
	consequences of early bathing
Cord care	Clean cord-cutting instrument and surface
	Application of antiseptic (chlorhexidine) to the umbilical cord
	Not applying substance other than antiseptic to the cord
Breastfeeding	Early (within 1 hour) and exclusive breastfeeding, avoiding prelacteal feeds
	Feeding of colostrums
	Not feeding foods in addition to breastmilk for the first 6 months of life
Oil massage	Use of oil with protective properties, such as sunflower oil, promote as ‘hot’ oil
	Not using mustard oil for massage, providing information on harmful characteristics of mustard oil
Post-delivery care	Use of postnatal visit to mother and child to advise on proper neonatal care, hygiene, breastfeeding, and information on immunizations and reproductive health

TBAs=Traditional birth attendants; TT=Tetanus toxoid

Other studies have provided evidence that community-based approaches to improve maternal and neonatal health (20,39) and training and integrating TBAs (37,40) can be effective. Given the current pervasiveness of deliveries in the home, the potential to work with TBAs to promote and provide available low-cost, low-tech and effective interventions that address immediate neonatal care should be further explored.

## ACKNOWLEDGEMENTS

This project was made possible through NICHD grants (No. HD 44004 and No. HD38753), USAID cooperative agreements (No. HRN-A-00-97-00015-00 and No. GHS-A-00-03-000019-00), and the Bill & Melinda Gates Foundation (No. 810-2054).
